# Stealth Proteins: In Silico Identification of a Novel Protein Family Rendering Bacterial Pathogens Invisible to Host Immune Defense

**DOI:** 10.1371/journal.pcbi.0010063

**Published:** 2005-11-18

**Authors:** Peter Sperisen, Christoph D Schmid, Philipp Bucher, Olav Zilian

**Affiliations:** 1 Swiss Institute of Bioinformatics, Epalinges, Switzerland; 2 Swiss Institute for Experimental Cancer Research, Epalinges, Switzerland; EMBL Heidelberg, Germany

## Abstract

There are a variety of bacterial defense strategies to survive in a hostile environment. Generation of extracellular polysaccharides has proved to be a simple but effective strategy against the host's innate immune system. A comparative genomics approach led us to identify a new protein family termed Stealth, most likely involved in the synthesis of extracellular polysaccharides. This protein family is characterized by a series of domains conserved across phylogeny from bacteria to eukaryotes. In bacteria, Stealth (previously characterized as SacB, XcbA, or WefC) is encoded by subsets of strains mainly colonizing multicellular organisms, with evidence for a protective effect against the host innate immune defense. More specifically, integrating all the available information about Stealth proteins in bacteria, we propose that Stealth is a D-hexose-1-phosphoryl transferase involved in the synthesis of polysaccharides. In the animal kingdom, Stealth is strongly conserved across evolution from social amoebas to simple and complex multicellular organisms, such as *Dictyostelium discoideum*, hydra, and human. Based on the occurrence of Stealth in most Eukaryotes and a subset of Prokaryotes together with its potential role in extracellular polysaccharide synthesis, we propose that metazoan Stealth functions to regulate the innate immune system. Moreover, there is good reason to speculate that the acquisition and spread of Stealth could be responsible for future epidemic outbreaks of infectious diseases caused by a large variety of eubacterial pathogens. Our in silico identification of a homologous protein in the human host will help to elucidate the causes of Stealth-dependent virulence. At a more basic level, the characterization of the molecular and cellular function of Stealth proteins may shed light on fundamental mechanisms of innate immune defense against microbial invasion.

## Synopsis

The immune system is a complex and highly developed system of specialized cells and organs that protects an organism against bacterial, parasitic, fungal, and viral infections. Broadly speaking, the different types of immune responses subdivide the immune system into two categories: innate (or nonadaptive) and adaptive immune system. The innate immune system serves as a first line of defense but lacks the ability to recognize certain pathogens and to provide the specific protective immunity that prevents reinfection. Just as metazoans have developed many different defenses against pathogens, so have pathogens evolved elaborate strategies to evade these defenses. Based on a comparative genomics approach and data mining, the authors have discovered a new family of proteins with a striking phylogenetic distribution, occurring in most eukaryotes and in subsets of mostly pathogenic or commensal prokaryotes. While the precise functions of these proteins remain unknown, prokaryotic versions have been implicated in the synthesis of extracellular polysaccharides known to be potent regulators of the innate immune system. This previously unrecognized link hints towards a potentially novel regulatory mechanism of the innate immune system. It remains to be shown if drugs selectively inhibiting Stealth in pathogens will help fight Stealth-mediated infections.

## Introduction

Colonization of hosts by microorganisms is a complex process that determines if the microorganism will coexist with the host as commensal, become an invasive pathogen, or be efficiently eliminated by the host's immune defense [1,2]. Consequently, microorganisms have developed a variety of measures to cope with the increasingly sophisticated defense strategies of the host's immune system [3–7]. Amongst them, the generation of an extracellular coat made of polysaccharides has proved to be a simple but effective strategy. Bacterial surface polysaccharides can be either amorphous exopolysaccharides, anchored in the lipid layer (lipopolysaccharides, another known regulator of the immune system), or organized as a capsule (capsule polysaccharides [CPSs]). The latter have been shown to mediate adherence to cells and, more importantly, protection against the host's innate immune system [8–11].

Different strategies to escape host immune surveillance have evolved through vertical evolution but also through horizontal gene transfer [12–15]. Though a subject of long-standing controversy, there is increasing evidence suggesting that horizontal gene transfer also occurs from eukaryotes to prokaryotes [16]. Even though the recombined bacteria seemed to have preferentially retained individual domains of proteins [16], a first example was recently reported in which certain bacterial strains kept an entire open reading frame [17].

Here we describe a novel protein family named “Stealth.” Based on a comparative genomics approach, we propose a biological function and an evolutionary scenario for this new protein family.

## Results/Discussion

### Identification of Stealth

In a screen of the human genome for Notch-related proteins, a novel protein containing two copies of Lin-12/Notch repeats was identified. The protein also showed strong sequence similarity to a number of animal and bacterial proteins, including several virulence factors of human pathogens published under different names. This previously unknown protein family was named “Stealth” because experimentally characterized members of this family appear to render bacterial and protozoan invaders invisible to the host's immune surveillance system.

**Figure 1:**
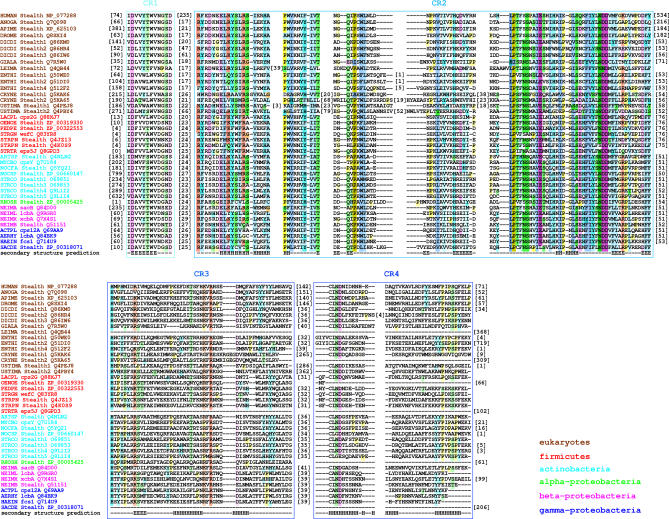
Multiple Alignments of CRs Multiple alignments of the four CRs for a representative set of protein sequences (>15% dissimilarity over all four CRs) are shown. Sequences are identified by a species code (see Table 1), protein name (from literature as proposed in this paper), and database accession number, where available. The lengths of the sequences omitted between or within CRs are indicated in square brackets. The last row shows the secondary structure prediction obtained by jnetpred [65] for the human Stealth protein, where H stands for helices and E for beta-sheets. The color scheme used is the ClustalX default scheme, with the colors for conserved amino acids being more intense than those for nonconserved ones.

**Figure 2:**
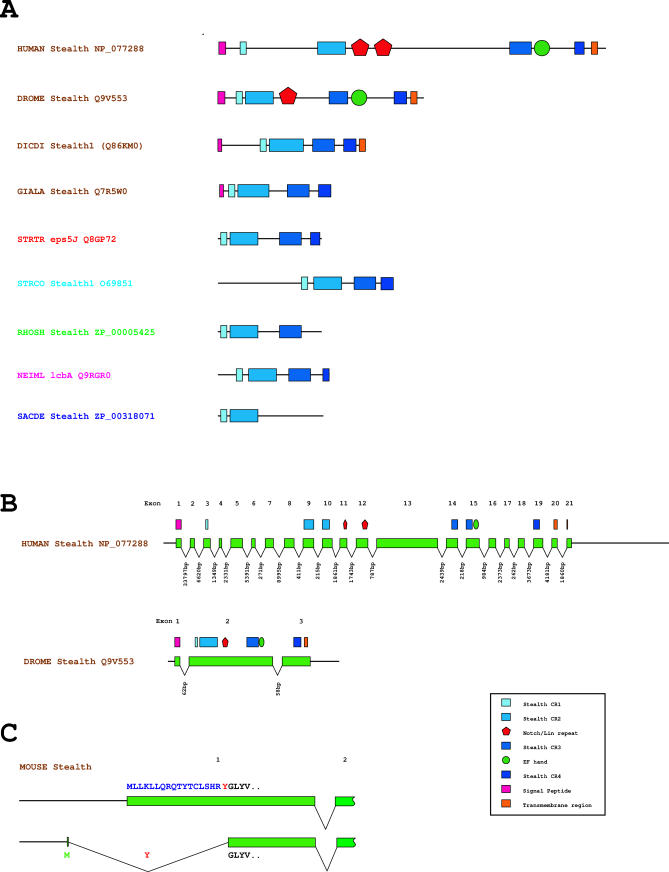
Domain Architecture and Genome Structure (A) CR1 to CR4, found through multiple alignments, are represented by rectangles ranging from light blue (CR1) to dark blue (CR4). Other motifs are represented as follows: predicted signal peptides as magenta rectangles, transmembrane regions as orange rectangles, Lin-12/Notch repeats as red pentagons, and EF-hands as green circles. (B) The genome structure of the human and fly Stealth homologs is represented, with the exons depicted as green rectangles separated by introns of indicated size. (C) Two splice variants lead to different N-terminal sequences, as supported by mouse EST sequences. Splicing reconstructs a codon for tyrosine (Y). Both proteins contain a predicted signal peptide.

Stealth proteins are characterized by four conserved regions (CRs) referred to as CR1 to CR4 (Figure 1). The N-terminal CR1 consists of a short but strongly conserved sequence motif, IDVVYTF or very similar. The second region, CR2, is approximately 100 residues long and constitutes the most conserved part of this protein family. A standard BLAST search [18] with any CR2 domain identifies all other members of the Stealth family in the current database with highly significant *E*-values. CR3 is about 50 residues long but less well conserved. Finally, the C-terminal CR4 includes an almost universally conserved tetrapetide, CLND or CIND. Adjacent and between these domains are divergent sequence regions of variable length that may contain additional domains (Figures 1 and 2A).

**Table 1:**
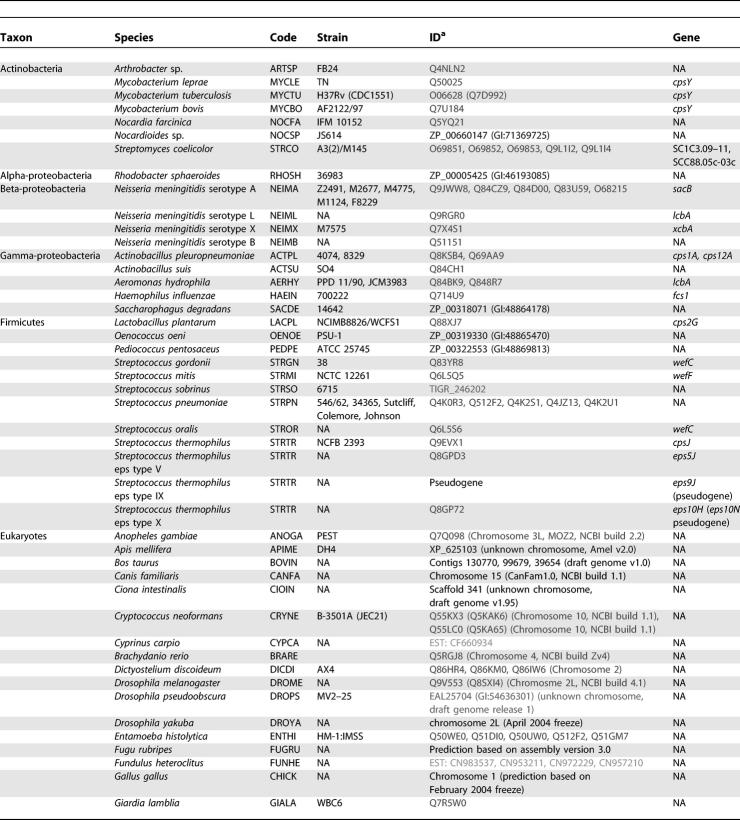
Summary of All Species Containing Stealth Proteins

**Table 1:**
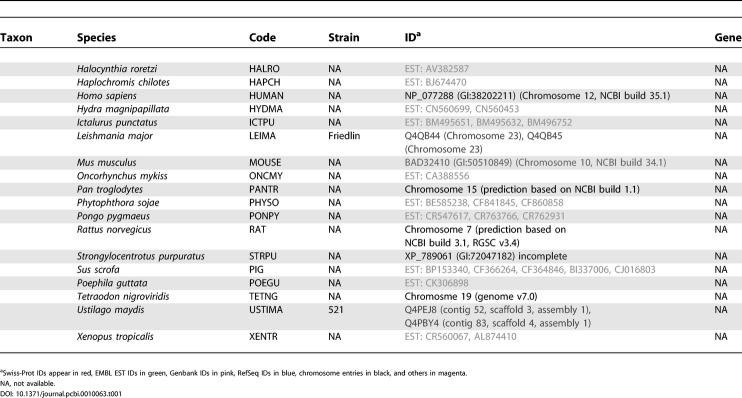
Continued

### Taxonomic Distribution

Stealth proteins are found encoded in the genomes of chordates, echinodermates, hydras, fungi, and flies but appear to be absent from nematodes and plants. Interestingly, a few organisms contain multiple Stealth genes (Table 1). Stealth proteins also occur in the protist genomes of *Dictyostelium, Giardia, Leishmania, Entamoeba,* and *Phytophthora,* and among the hitherto sequenced bacteria, they are found in the following phyla: alpha-, beta-, and gamma-proteobacteria (mostly pathogens), firmicutes (mostly the commensals), and actinobacteria (some animal pathogens) (Table 1; Figure S1). It is noteworthy that the large majority of completely sequenced bacterial genomes do not harbor Stealth. The species that do contain a member of this family are not necessarily closely related, and include Gram-positive as well as Gram-negative bacteria.

### Stealth in Bacteria

Several of the documented bacterial Stealth genes belong to capsule group II biosynthesis operons generating carbohydrate-phosphodiester-containing CPSs [19–24]. In the case of Stealth-expressing bacteria, these CPSs turned out to inhibit complement-mediated lysis, as shown for serogroup A and X of *Neisseria meningitidis* [23,24] and to correlate with serum and phagocyte survival abilities as shown for *Aeromonas hydrophila* [25].

The majority of Stealth-expressing bacteria that have been analyzed so far for the composition of their exopolysaccharides turned out to build phosphoglycans consisting of phosphodiester-linked hexose mono- or disaccharide building blocks [26–29]. On the other hand, certain bacteria living in a biofilm community contain CPSs consisting of phosphodiester-linked hexa- or heptasaccharide repeating units [30,31]. These carbohydrates, also called receptor polysaccharides, are synthesized by a series of different glycosyltransferases, with Stealth amongst them [22]. Strains encoding Stealth carry a hexose phosphodiester linker [31] in their receptor polysaccharides, whereas strains lacking Stealth build receptor polysaccharides with a pentose phosphodiester linker.

Definite proof for an essential function of Stealth in CPS biosynthesis was shown in *N. meningitidis* serogroup A by selective deletion of the gene *sacB* (i.e., Stealth), giving rise to virtually unencapsulated mutants [23], and by deletion of part of the gene *xcbA* (i.e., Stealth), together with flanking open reading frames in a serogroup X strain, which resulted in complement-sensitive mutants [24]. Moreover, when the gene *cps1A* (i.e., Stealth) was deleted in *Actinobacillus pleuropneumoniae,* the resulting strains lost their pathogenicity in pigs [20].

Taken together, all of the above data suggest that Stealth is a D-hexose-1-phosphoryl transferase that generates interglycosidic phosphate diester linkages.

### Characteristics of Metazoan Stealth

Unlike the bacterial Stealth proteins, the vertebrate members of this family are not properly represented in current protein databases. We have manually reconstructed the gene and protein sequences for a number of species with the aid of EST sequences and cross-genome comparisons (Table 1). The human gene consists of 21 exons (Figure 2B), and the translated protein sequence is identical to the RefSeq entry NP_077288. The intron–exon structures of genes found in other vertebrates are essentially the same. In the mouse, however, there is a facultative intron near the start codon spliced out predominantly in transcripts from dendritic cells. This alternative splicing leads to two protein variants with different N-termini (Figure 2C). The hypothetical *Drosophila melanogaster* and *D. yakuba* Stealth genes, however, have a completely different intron–exon structure (Figure 2B). Finally, pieces of Stealth-encoding sequences were also found in the preliminary genomes or ESTs of other mammals (Table 1).

Metazoan Stealth proteins are characterized by additional domains. There is a predicted signal peptide and, near the C-terminus, a transmembrane helix. One or two Notch/Lin-12 repeats [32] are inserted between CR2 and CR3, and an EF-hand domain [33] appears between CR3 and CR4. So far, all reconstructed Stealth proteins contain these domains, and in some of the cases where only pieces of sequences are available one can identify these motifs. The strong conservation of the Stealth domain architecture suggests that this protein plays an essential role.

No experimental knowledge is available about the function of metazoan Stealth proteins today (note, however, that Stealth-deficient mice have been generated by O. Z. and coworkers and will be made available upon request). In view of the high degree of sequence similarity to their bacterial homologs, it is reasonable to speculate that they have a similar molecular function and thus are also implicated in exopolysaccharide synthesis. Public expression profiles derived from SAGE experiments indicate a rather broad tissue distribution. The Stealth-dependent polysaccharides could be host-specific structural surface elements exploited by the immune system for self-recognition. In this case, the Stealth-dependent resistance of human pathogens to complement-mediated lysis and other host defense mechanisms would be a straightforward case of molecular mimicry. Alternatively, host-encoded Stealth proteins may play an active role in down-regulating the immune response. The presence of Stealth in both insects and urochordates further suggests that this protein interferes with processes related to innate rather than adaptive immunity [34,35].

### Stealth and Protists

Although higher eukaryotes haven't yet been investigated for the presence of phosphoglycan structures similar to the CPSs, such structures have been identified in *D. discoideum* and in *Leishmania* species. In *D. discoideum* such polysaccharides were found on lysosomal cysteine proteinases and spore coat proteins [36,37]. The lysosomal enzymes of *D. discoideum* have two types of carbohydrate modifications [38,39] found in two separate sets of lysosomal vesicles [40,41]. The major component of *Leishmania* lipophosphoglycan is a heteropolymer of 10–40 phosphodiester-linked disaccharide units, depending on species and developmental stage [42]. Lipophosphoglycan is predominantly expressed by promastigotes, is essential for intracellular survival in macrophages and for the virulence of *Leishmania major* and *L. donovani,* and disappears when the pathogen intracellularly differentiates into amastigotes within host phagolysosomes [43–47]. The genes encoding these hexose-phosphoryl transferases have been identified neither in *D. discoideum* nor in *Leishmania*. Given, however, Stealth's presumed enzymatic activity and its comparative biochemical characterization from three different *Leishmania* species using synthetic acceptor substrate analogs [48], the two Stealth proteins found in *Leishmania* and those found in *D. discoideum* are good candidates for this function.

### Evolution of Stealth

**Figure 3:**
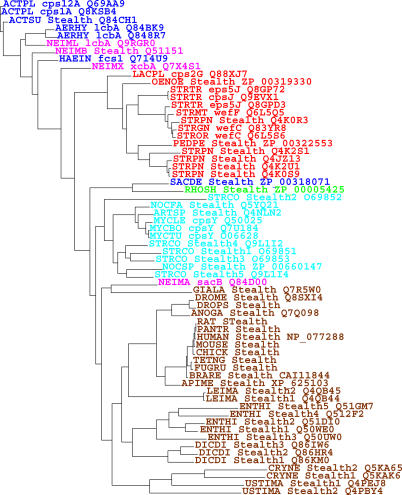
Phylogenetic Tree Trees were calculated from amino acid sequence alignments of the four CRs. As in Figure 1, sequences are identified by a species code (see Table 1), protein name (from literature as proposed in this paper), and database accession number, and are color-coded. Dissimilarities are represented by the length of the branches (all with posterior probabilities above 0.95).

The peculiar taxonomic distribution of Stealth (Figure 3) could be the outcome of two different evolutionary scenarios: (i) differential loss of an ancient protein already present in an ancestral form of life, or (ii) horizontal gene transfer between eukaryotes and eubacteria. The second hypothesis appears to be the more plausible, but the direction of the transfer is more difficult to assess. Overall, the protein tree largely follows species phylogeny, at least with regard to the higher level taxonomic groups. This indicates that transfer between eukaryotes and prokaryotes must have been an ancient event. However, several observations suggest that Stealth proteins continue to be horizontally transferred within and between certain bacterial groups. In Gram-negative bacteria, Stealth is inserted into group II capsule operons, which exhibit strong sequence similarity across many species, thus facilitating horizontal gene transfer via homologous recombination [49,50]. Moreover, certain Stealth genes have significantly lower G+C content than the remaining part of the genome [19,21,24,51], which is indicative of a recent acquisition from another species, and some of these genes are flanked by recombination-promoting IS insertion elements or residual fragments thereof [21,24].

## Materials and Methods

### Sequence analysis.

Multiple amino acid sequence alignments of the four CRs were generated using T-Coffee [52]. The signal peptides were predicted with SignalP v2.0 using the combined NN/HMM-based method [53,54], the transmembrane predictions were made using TMHMM v2.0 [55,56], and the Lin-12/Notch repeats were identified using the profile PS50258 in PROSITE [57]. The EF-hand domains were detected using the Pfam HMM PF00036 [58].

The human and the fly gene structures were constructed with the aid of the trome database [59–61].

### Sequence database searches.

Other members of the Stealth protein family were identified by searching with either the human or the *Streptomyces coelicolor* CR2 using BLAST [18] on either nucleic acid or protein databases.

### Calculation of sequence trees.

For each CR a separate multiple amino acid sequence alignment was generated. These multiple alignments were concatenated, resulting in a multiple alignment that represents the four CRs. CRs that are absent in certain species are represented as gaps in the multiple alignment. Processed alignments were used to derive tree topologies using Bayesian inference of phylogeny as implemented by MrBayes v3.0 [62,63]. MrBayes was used with four heated chains over 200,000 generations, sampling every 20 trees. The likelihoods of these trees were examined to estimate the length of the burn-in phase, and all trees sampled 20,000 generations later than this point were used to create a consensus tree using the 50% majority rule. MrBayes was used with the mixed model of amino acid substitution, assuming the presence of invariant sites and using a gamma distribution approximated by four different rate categories to model rate variation between sites, estimating amino acid frequencies from the alignment. The consensus tree was displayed using DRAWGRAM of the PHYLIP package [64].

## Supporting Information

Figure S1 Taxonomic Distribution of Stealth in Bacteria (57 KB DOC)

## Abbreviations

CPS: capsule polysaccharide
CR: conserved region

## References

[pcbi-0010063-b001] Merrell DS, Falkow S (2004). Frontal and stealth attack strategies in microbial pathogenesis. Nature.

[pcbi-0010063-b002] Waterfield NR, Wren BW, Ffrench-Constant RH (2004). Invertebrates as a source of emerging human pathogens. Nat Rev Microbiol.

[pcbi-0010063-b003] Irving P, Troxler L, Hetru C (2004). Is innate enough? The innate immune response in *Drosophila*. C R Biol.

[pcbi-0010063-b004] Brown DR (2002). Mycoplasmosis and immunity of fish and reptiles. Front Biosci.

[pcbi-0010063-b005] Kalia A, Bessen DE (2004). Natural selection and evolution of streptococcal virulence genes involved in tissue-specific adaptations. J Bacteriol.

[pcbi-0010063-b006] Claverys JP, Prudhomme M, Mortier-Barriere I, Martin B (2000). Adaptation to the environment: *Streptococcus pneumoniae,* a paradigm for recombination-mediated genetic plasticity?. Mol Microbiol.

[pcbi-0010063-b007] Fleckenstein JM, Kopecko DJ (2001). Breaching the mucosal barrier by stealth: An emerging pathogenic mechanism for enteroadherent bacterial pathogens. J Clin Invest.

[pcbi-0010063-b008] Costerton JW, Cheng KJ, Geesey GG, Ladd TI, Nickel JC (1987). Bacterial biofilms in nature and disease. Annu Rev Microbiol.

[pcbi-0010063-b009] Roberts IS, Saunders FK, Boulnois GJ (1989). Bacterial capsules and interactions with complement and phagocytes. Biochem Soc Trans.

[pcbi-0010063-b010] Cross AS (1990). The biologic significance of bacterial encapsulation. Curr Top Microbiol Immunol.

[pcbi-0010063-b011] Moxon ER, Kroll JS (1990). The role of bacterial polysaccharide capsules as virulence factors. Curr Top Microbiol Immunol.

[pcbi-0010063-b012] Lorenz MG, Wackernagel W (1994). Bacterial gene transfer by natural genetic transformation in the environment. Microbiol Rev.

[pcbi-0010063-b013] Hartl DL, Dykhuizen DE, Berg DE (1984). Accessory DNAs in the bacterial gene pool: Playground for coevolution. Ciba Found Symp.

[pcbi-0010063-b014] Moreira D (2000). Multiple independent horizontal transfers of informational genes from bacteria to plasmids and phages: Implications for the origin of bacterial replication machinery. Mol Microbiol.

[pcbi-0010063-b015] Sundstrom L (1998). The potential of integrons and connected programmed rearrangements for mediating horizontal gene transfer. APMIS Suppl.

[pcbi-0010063-b016] Koonin EV, Makarova KS, Aravind L (2001). Horizontal gene transfer in prokaryotes: Quantification and classification. Annu Rev Microbiol.

[pcbi-0010063-b017] Budd A, Blandin S, Levashina EA, Gibson TJ (2004). Bacterial alpha2-macroglobulins: Colonization factors acquired by horizontal gene transfer from the metazoan genome?. Genome Biol.

[pcbi-0010063-b018] Altschul SF, Gish W, Miller W, Myers EW, Lipman DJ (1990). Basic local alignment search tool. J Mol Biol.

[pcbi-0010063-b019] Satola SW, Schirmer PL, Farley MM (2003). Genetic analysis of the capsule locus of *Haemophilus influenzae* serotype f. Infect Immun.

[pcbi-0010063-b020] Bandara AB, Lawrence ML, Veit HP, Inzana TJ (2003). Association of *Actinobacillus pleuropneumoniae* capsular polysaccharide with virulence in pigs. Infect Immun.

[pcbi-0010063-b021] Almiron-Roig E, Mulholland F, Gasson MJ, Griffin AM (2000). The complete cps gene cluster from *Streptococcus thermophilus* NCFB 2393 involved in the biosynthesis of a new exopolysaccharide. Microbiology.

[pcbi-0010063-b022] Xu DQ, Thompson J, Cisar JO (2003). Genetic loci for coaggregation receptor polysaccharide biosynthesis in *Streptococcus gordonii* 38. J Bacteriol.

[pcbi-0010063-b023] Swartley JS, Liu LJ, Miller YK, Martin LE, Edupuganti S (1998). Characterization of the gene cassette required for biosynthesis of the (alpha1→6)-linked N-acetyl-D-mannosamine-1-phosphate capsule of serogroup A *Neisseria meningitidis*. J Bacteriol.

[pcbi-0010063-b024] Tzeng YL, Noble C, Stephens DS (2003). Genetic basis for biosynthesis of the (alpha 1→4)-linked N-acetyl-D-glucosamine 1-phosphate capsule of *Neisseria meningitidis* serogroup X. Infect Immun.

[pcbi-0010063-b025] Zhang YL, Lau YL, Arakawa E, Leung KY (2003). Detection and genetic analysis of group II capsules in *Aeromonas hydrophila*. Microbiology.

[pcbi-0010063-b026] Liu TY, Gotschlich EC, Dunne FT, Jonssen EK (1971). Studies on the meningococcal polysaccharides. II. Composition and chemical properties of the group B and group C polysaccharide. J Biol Chem.

[pcbi-0010063-b027] Liu TY, Gotschlich EC, Jonssen EK, Wysocki JR (1971). Studies on the meningococcal polysaccharides. I. Composition and chemical properties of the group A polysaccharide. J Biol Chem.

[pcbi-0010063-b028] Bundle DR, Jennings HJ, Kenny CP (1974). Studies on the group-specific polysaccharide of *Neisseria meningitidis* serogroup X and an improved procedure for its isolation. J Biol Chem.

[pcbi-0010063-b029] Branefors-Helander P, Kenne L, Lindqvist B (1980). Structural studies of the capsular antigen from *Haemophilus influenzae* type f. Carbohydr Res.

[pcbi-0010063-b030] Reddy GP, Abeygunawardana C, Bush CA, Cisar JO (1994). The cell wall polysaccharide of *Streptococcus gordonii* 38: Structure and immunochemical comparison with the receptor polysaccharides of *Streptococcus oralis* 34 and *Streptococcus mitis* J22. Glycobiology.

[pcbi-0010063-b031] Cisar JO, Sandberg AL, Reddy GP, Abeygunawardana C, Bush CA (1997). Structural and antigenic types of cell wall polysaccharides from viridans group streptococci with receptors for oral actinomyces and streptococcal lectins. Infect Immun.

[pcbi-0010063-b032] Vardar D, North CL, Sanchez-Irizarry C, Aster JC, Blacklow SC (2003). Nuclear magnetic resonance structure of a prototype Lin12-Notch repeat module from human Notch1. Biochemistry.

[pcbi-0010063-b033] Ikura M (1996). Calcium binding and conformational response in EF-hand proteins. Trends Biochem Sci.

[pcbi-0010063-b034] Cooper EL (2003). Comparative immunology. Curr Pharm Des.

[pcbi-0010063-b035] Khalturin K, Panzer Z, Cooper MD, Bosch TC (2004). Recognition strategies in the innate immune system of ancestral chordates. Mol Immunol.

[pcbi-0010063-b036] Srikrishna G, Wang L, Freeze HH (1998). Fucosebeta-1-P-Ser is a new type of glycosylation: Using antibodies to identify a novel structure in *Dictyostelium discoideum* and study multiple types of fucosylation during growth and development. Glycobiology.

[pcbi-0010063-b037] Mreyen M, Champion A, Srinivasan S, Karuso P, Williams KL (2000). Multiple O-glycoforms on the spore coat protein SP96 in *Dictyostelium discoideum*. Fuc(alpha1–3)GlcNAc-alpha-1-P-Ser is the major modification. J Biol Chem.

[pcbi-0010063-b038] Freeze HH, Montreuil J, Vliegenhart JFG, Schachter H (1997). *Dictyostelium discoideum* glycoproteins: Using a model system for organismic glycobiology. Glycoproteins II.

[pcbi-0010063-b039] Gustafson GL, Gander JE (1984). O beta-(N-acetyl-alpha-glucosamine-1-phosphoryl)serine in proteinase I from *Dictyostelium discoideum*. Methods Enzymol.

[pcbi-0010063-b040] Mehta DP, Ichikawa M, Salimath PV, Etchison JR, Haak R (1996). A lysosomal cysteine proteinase from *Dictyostelium discoideum* contains N-acetylglucosamine-1-phosphate bound to serine but not mannose-6-phosphate on N-linked oligosaccharides. J Biol Chem.

[pcbi-0010063-b041] Souza GM, Mehta DP, Lammertz M, Rodriguez-Paris J, Wu R (1997). *Dictyostelium* lysosomal proteins with different sugar modifications sort to functionally distinct compartments. J Cell Sci.

[pcbi-0010063-b042] Descoteaux A, Turco SJ (1999). Glycoconjugates in *Leishmania* infectivity. Biochim Biophys Acta.

[pcbi-0010063-b043] McNeely TB, Turco SJ (1990). Requirement of lipophosphoglycan for intracellular survival of *Leishmania donovani* within human monocytes. J Immunol.

[pcbi-0010063-b044] Dermine JF, Scianimanico S, Prive C, Descoteaux A, Desjardins M (2000). *Leishmania* promastigotes require lipophosphoglycan to actively modulate the fusion properties of phagosomes at an early step of phagocytosis. Cell Microbiol.

[pcbi-0010063-b045] Scianimanico S, Desrosiers M, Dermine JF, Meresse S, Descoteaux A (1999). Impaired recruitment of the small GTPase rab7 correlates with the inhibition of phagosome maturation by *Leishmania donovani* promastigotes. Cell Microbiol.

[pcbi-0010063-b046] Spath GF, Lye LF, Segawa H, Sacks DL, Turco SJ (2003). Persistence without pathology in phosphoglycan-deficient *Leishmania major*. Science.

[pcbi-0010063-b047] Spath GF, Garraway LA, Turco SJ, Beverley SM (2003). The role(s) of lipophosphoglycan (LPG) in the establishment of *Leishmania major* infections in mammalian hosts. Proc Natl Acad Sci U S A.

[pcbi-0010063-b048] Routier FH, Higson AP, Ivanova IA, Ross AJ, Tsvetkov YE (2000). Characterization of the elongating alpha-D-mannosyl phosphate transferase from three species of *Leishmania* using synthetic acceptor substrate analogues. Biochemistry.

[pcbi-0010063-b049] Coffey TJ, Dowson CG, Daniels M, Zhou J, Martin C (1991). Horizontal transfer of multiple penicillin-binding protein genes, and capsular biosynthetic genes, in natural populations of *Streptococcus pneumoniae*. Mol Microbiol.

[pcbi-0010063-b050] Kroll JS, Moxon ER (1990). Capsulation in distantly related strains of *Haemophilus influenzae* type b: Genetic drift and gene transfer at the capsulation locus. J Bacteriol.

[pcbi-0010063-b051] Tettelin H, Saunders NJ, Heidelberg J, Jeffries AC, Nelson KE (2000). Complete genome sequence of *Neisseria meningitidis* serogroup B strain MC58. Science.

[pcbi-0010063-b052] Notredame C, Higgins DG, Heringa J (2000). T-Coffee: A novel method for fast and accurate multiple sequence alignment. J Mol Biol.

[pcbi-0010063-b053] Nielsen H, Engelbrecht J, Brunak S, von Heijne G (1997). Identification of prokaryotic and eukaryotic signal peptides and prediction of their cleavage sites. Protein Eng.

[pcbi-0010063-b054] Nielsen H, Krogh A (1998). Prediction of signal peptides and signal anchors by a hidden Markov model. Proc Int Conf Intell Syst Mol Biol.

[pcbi-0010063-b055] Krogh A, Larsson B, von Heijne G, Sonnhammer EL (2001). Predicting transmembrane protein topology with a hidden Markov model: Application to complete genomes. J Mol Biol.

[pcbi-0010063-b056] Sonnhammer EL, von Heijne G, Krogh A (1998). A hidden Markov model for predicting transmembrane helices in protein sequences. Proc Int Conf Intell Syst Mol Biol.

[pcbi-0010063-b057] Hofmann K, Bucher P, Falquet L, Bairoch A (1999). The PROSITE database, its status in 1999. Nucleic Acids Res.

[pcbi-0010063-b058] Bateman A, Birney E, Durbin R, Eddy SR, Finn RD (1999). Pfam 3.1: 1313 multiple alignments and profile HMMs match the majority of proteins. Nucleic Acids Res.

[pcbi-0010063-b059] Iseli C, Stevenson BJ, de Souza SJ, Samaia HB, Camargo AA (2002). Long-range heterogeneity at the 3′ ends of human mRNAs. Genome Res.

[pcbi-0010063-b060] Stevenson BJ, Iseli C, Beutler B, Jongeneel CV (2003). Use of transcriptome data to unravel the fine structure of genes involved in sepsis. J Infect Dis.

[pcbi-0010063-b061] Sperisen P, Iseli C, Pagni M, Stevenson BJ, Bucher P (2004). trome, trEST and trGEN: Databases of predicted protein sequences. Nucleic Acids Res.

[pcbi-0010063-b062] Huelsenbeck JP, Ronquist F (2001). MRBAYES: Bayesian inference of phylogenetic trees. Bioinformatics.

[pcbi-0010063-b063] Ronquist F, Huelsenbeck JP (2003). MrBayes 3: Bayesian phylogenetic inference under mixed models. Bioinformatics.

[pcbi-0010063-b064] Felsenstein J (1989). PHYLIP—Phylogeny Inference Package (version 3.2). Cladistics.

[pcbi-0010063-b065] Cuff JA, Barton GJ (1999). Evaluation and improvement of multiple sequence methods for protein secondary structure prediction. Proteins.

