# Supplementary material for: Stealth Proteins: In Silico Identification of a Novel Protein Family Rendering Bacterial Pathogens Invisible to Host Immune Defense
Source: PLoS Comput Biol. 2005 Nov 18;1(6):e63. doi: 10.1371/journal.pcbi.0010063 (PMC1285062; doi:10.1371/journal.pcbi.0010063)
Supplement: Supplementary file 1 [file pcbi.0010063.sg001.doc]

**Supporting Information**

Figure S1. Taxonomic distribution of Stealth in bacteria.

Pseudotree of all bacteria with known Stealth status. The taxonomy-based tree includes as leaves all species and strains for which there is a completely sequenced genome or positive evidence of a *stealth* gene. Strains containing Stealth are marked in red.

```
--|-------------Corynebacterium diphtheriae NCTC 13129
  |   | | | |   |-Corynebacterium efficiens YS-314
  |   | | | |   |-Corynebacterium glutamicum ATCC 13032
  |   | | | |   |-Corynebacterium jeikeium K411
  |   | | | |-----------Mycobacterium avium subsp. paratuberculosis str. k10
  |   | | | |   |-Mycobacterium leprae TN
  |   | | | |   |---Mycobacterium bovis AF2122/97
  |   | | | |     |-Mycobacterium tuberculosis CDC1551
  |   | | | |     |-Mycobacterium tuberculosis H37Rv
  |   | | | |-----Nocardia farcinica IFM 10152
  |   | | |-------Kineococcus radiotolerans SRS30216
  |   | | |-------Brevibacterium linens BL2
  |   | | | |-----Tropheryma whipplei str. Twist
  |   | | | |   |-Tropheryma whipplei TW08/27
  |   | | | |---------Leifsonia xyli subsp. xyli str. CTCB07
  |   | | | |-----Arthrobacter sp.
  |   | | |-------Nocardioides sp.
  |   | | | |-----Propionibacterium acnes KPA171202
  |   | | |-------Streptomyces avermitilis MA-4680
  |   | | |     |-Streptomyces coelicolor A3(2)
  |   | | |-------Thermobifida fusca YX
  |   | |-------Bifidobacterium longum DJO10A
  |   |       |-Bifidobacterium longum NCC2705
  |   |-----------Rubrobacter xylanophilus DSM 9941
  |   |-----Symbiobacterium thermophilum IAM 14863
  |-----------Aquifex aeolicus VF5
  |-------------Bacteroides fragilis NCTC 9343
  | | |   |   |-Bacteroides fragilis YCH46
  | | |   |   |-Bacteroides thetaiotaomicron VPI-5482
  | | |   |-----Porphyromonas gingivalis W83
  | | |---------Cytophaga hutchinsonii
  | |-------------Chlorobium tepidum TLS
  |-------------Chlamydia muridarum Nigg
  |       | | |-Chlamydia trachomatis D/UW-3/CX
  |       | |---Chlamydophila abortus S26/3
  |       |   |-Chlamydophila caviae GPIC
  |       |   |-Chlamydophila pneumoniae AR39
  |       |   |-Chlamydophila pneumoniae CWL029
  |       |   |-Chlamydophila pneumoniae J138
  |       |   |-Chlamydophila pneumoniae TW-183
  |       |-----Parachlamydia sp. UWE25
  |-----------Chloroflexus aurantiacus
  | |-----Dehalococcoides ethenogenes 195
  |     |-Dehalococcoides sp. CBDB1
  |-------Crocosphaera watsonii WH 8501
  | | |---Synechococcus elongatus PCC 6301
  | | | |-Synechococcus elongatus PCC 7942
  | | | |-Synechococcus sp. WH 8102
  | | |---Synechocystis sp. PCC 6803
  | | |---Thermosynechococcus elongatus BP-1
  | |-------Gloeobacter violaceus PCC 7421
  | |-------Anabaena variabilis ATCC 29413
  | |   |---Nostoc punctiforme PCC 73102
  | |     |-Nostoc sp. PCC 7120
  | |-----Trichodesmium erythraeum IMS101
  | |-------Prochlorococcus marinus str. MIT 9313
  |       |-Prochlorococcus marinus str. NATL2A
  |       |-----Prochlorococcus marinus subsp. marinus str. CCMP1375
  |         |---Prochlorococcus marinus subsp. pastoris str. CCMP1986
  |-----------Deinococcus radiodurans R1
  |   |-------Thermus thermophilus HB27
  |         |-Thermus thermophilus HB8
  |-----------Pasteuria nishizawae str. North American
  | | | |-------Bacillus anthracis str. A1055
  | | | | | | |-Bacillus anthracis str. A2012
  | | | | | | |-Bacillus anthracis str. Ames
  | | | | | | |-Bacillus anthracis str. 'Ames Ancestor'
  | | | | | | |-Bacillus anthracis str. Australia 94
  | | | | | | |-Bacillus anthracis str. CNEVA-9066
  | | | | | | |-Bacillus anthracis str. Kruger B
  | | | | | | |-Bacillus anthracis str. Sterne
  | | | | | | |-Bacillus anthracis str. Vollum
  | | | | | | |-Bacillus anthracis str. Western North America USA6153
  | | | | | | |-Bacillus cereus ATCC 10987
  | | | | | | |-Bacillus cereus ATCC 14579
  | | | | | | |-Bacillus cereus E33L
  | | | | | | |-Bacillus cereus G9241
  | | | | | | |-----Bacillus thuringiensis serovar konkukian str. 97-27
  | | | | | |-Bacillus clausii KSM-K16
  | | | | | |-Bacillus halodurans C-125
  | | | | | |-Bacillus licheniformis ATCC 14580
  | | | | | |-----Bacillus subtilis subsp. subtilis str. 168
  | | | | |---Exiguobacterium sp. 255-15
  | | | | |---Geobacillus kaustophilus HTA426
  | | | | |---Oceanobacillus iheyensis HTE831
  | | | |-----Listeria innocua Clip11262
  | | | |   |-Listeria monocytogenes EGD-e
  | | | |   |-Listeria monocytogenes str. 1/2a F6854
  | | | |   |-Listeria monocytogenes str. 4b F2365
  | | | |   |-Listeria monocytogenes str. 4b H7858
  | | | |---------Staphylococcus aureus subsp. aureus COL
  | | |     |   |-Staphylococcus aureus subsp. aureus MRSA252
  | | |     |   |-Staphylococcus aureus subsp. aureus MSSA476
  | | |     |   |-Staphylococcus aureus subsp. aureus Mu50
  | | |     |   |-Staphylococcus aureus subsp. aureus MW2
  | | |     |   |-Staphylococcus aureus subsp. aureus N315
  | | |     |-Staphylococcus epidermidis ATCC 12228
  | | |     |-Staphylococcus epidermidis RP62A
  | | |     |-Staphylococcus haemolyticus JCSC1435
  | | |     |-----Staphylococcus saprophyticus subsp. saprophyticus ATCC 15305
  | | |-------Enterococcus faecalis V583
  | |   |   |-Enterococcus faecium
  | |   |-----Lactobacillus acidophilus NCFM
  | |   | | |-Lactobacillus casei ATCC 334
  | |   | | |-----Lactobacillus delbrueckii subsp. bulgaricus ATCC BAA-365
  | |   | | |-Lactobacillus gasseri
  | |   | | |-Lactobacillus johnsonii NCC 533
  | |   | | |-Lactobacillus plantarum WCFS1
  | |   | |---Pediococcus pentosaceus ATCC 25745
  | |   |---------Leuconostoc mesenteroides subsp. mesenteroides ATCC 8293
  | |   | |---Oenococcus oeni PSU-1
  | |   |---------Lactococcus lactis subsp. cremoris SK11
  | |     |   |---Lactococcus lactis subsp. lactis Il1403
  | |     |-------Streptococcus agalactiae NEM316
  | |       | |---Streptococcus agalactiae 2603V/R
  | |       |-Streptococcus gordonii 38
  | |       |-Streptococcus mitis NCTC 12261
  | |       |-Streptococcus mutans UA159
  | |       |-Streptococcus oralis (unknown strain)
  | |       |-Streptococcus pneumoniae R6
  | |       |-Streptococcus pneumoniae TIGR4
  | |       |-Streptococcus pneumoniae 546/62
  | |       |-Streptococcus pneumoniae 34365
  | |       |-Streptococcus pneumoniae Sutcliff
  | |       |-Streptococcus pneumoniae Colemore
  | |       |-Streptococcus pneumoniae Johnson
  | |       |-----Streptococcus pyogenes MGAS8232
  | |       | |---Streptococcus pyogenes M1 GAS
  | |       | | |-Streptococcus pyogenes MGAS5005
  | |       | |---Streptococcus pyogenes MGAS6180
  | |       | |---Streptococcus pyogenes MGAS315
  | |       | | |-Streptococcus pyogenes SSI-1
  | |       | |---Streptococcus pyogenes M49 591
  | |       | |---Streptococcus pyogenes MGAS10394
  | |       |-Streptococcus sobrinus 6715
  | |       |-Streptococcus suis 89/1591
  | |       |-Streptococcus thermophilus CNRZ1066
  | |       |-Streptococcus thermophilus LMD-9
  | |       |-Streptococcus thermophilus LMG 18311
  | |       |-Streptococcus thermophilus NCFB 2393
  | |       |-Streptococcus thermophilus (unknown strain)
  | |---------Clostridium acetobutylicum ATCC 824
  | | | |   |-Clostridium perfringens str. 13
  | | | |   |-Clostridium tetani E88
  | | | |   |-Clostridium thermocellum ATCC 27405
  | | | |-----Desulfitobacterium hafniense
  | | |---------Moorella thermoacetica ATCC 39073
  | |     |---Thermoanaerobacter tengcongensis MB4
  | |-------------Onion yellows phytoplasma OY-M
  |   |-------Mesoplasma florum L1
  |   |-------Mycoplasma gallisepticum R
  |       | |-Mycoplasma genitalium G-37
  |       | |-Mycoplasma hyopneumoniae 232
  |       | |-Mycoplasma hyopneumoniae 7448
  |       | |-Mycoplasma hyopneumoniae J
  |       | |-Mycoplasma mobile 163K
  |       | |-------Mycoplasma mycoides subsp. mycoides SC str. PG1
  |       | |-Mycoplasma penetrans HF-2
  |       | |-Mycoplasma pneumoniae M129
  |       | |-Mycoplasma pulmonis UAB CTIP
  |       | |-Mycoplasma synoviae 53
  |       |-------Ureaplasma parvum serovar 3 str. ATCC 700970
  |---------------Fusobacterium nucleatum subsp. nucleatum ATCC 25586
  |           |---Fusobacterium nucleatum subsp. vincentii ATCC 49256
  |-----------Rhodopirellula baltica SH 1
  |-------------Caulobacter crescentus CB15
  | | |-------Bartonella henselae str. Houston-1
  | | | |   |-Bartonella quintana str. Toulouse
  | | | |-----Bradyrhizobium japonicum USDA 110
  | | | | |---Rhodopseudomonas palustris CGA009
  | | | |-----Brucella melitensis 16M
  | | | |   |-------Brucella abortus biovar 1 str. 9-941
  | | | |     |---Brucella suis 1330
  | | | |-----Mesorhizobium loti MAFF303099
  | | | |   |-Mesorhizobium sp. BNC1
  | | | |-------Agrobacterium tumefaciens str. C58
  | | |   |-----Sinorhizobium meliloti 1021
  | | |-------Rhodobacter sphaeroides 36983
  | | |   |---Silicibacter pomeroyi DSS-3
  | | |     |-Silicibacter sp. TM1040
  | | |-------Gluconobacter oxydans 621H
  | | | |-----Magnetospirillum magnetotacticum
  | | |   |---Rhodospirillum rubrum
  | | |-------Anaplasma marginale str. St. Maries
  | | | | |-----Ehrlichia canis str. Jake
  | | | |     |-Ehrlichia ruminantium str. Gardel
  | | | |     |-Ehrlichia ruminantium str. Welgevonden
  | | | |---------Rickettsia akari str. Hartford
  | | | | |   | |-Rickettsia conorii str. Malish 7
  | | | | |   | |-Rickettsia felis URRWXCal2
  | | | | |   | |-Rickettsia rickettsii
  | | | | |   | |---Rickettsia sibirica 246
  | | | | |   |---Rickettsia prowazekii str. Madrid E
  | | | | |     |-Rickettsia typhi str. Wilmington
  | | | | |-------Wolbachia endosymbiont strain TRS of Brugia malayi
  | | | |     |-Wolbachia endosymbiont of Drosophila ananassae
  | | | |     |-Wolbachia endosymbiont of Drosophila melanogaster
  | | | |     |-Wolbachia endosymbiont of Drosophila simulans
  | | | |-------Candidatus Pelagibacter ubique HTCC1062
  | | |-------Erythrobacter litoralis HTCC2594
  | |   |-----Novosphingobium aromaticivorans DSM 12444
  | |     |-------Zymomonas mobilis subsp. mobilis ZM4
  | |---------Bordetella bronchiseptica RB50
  | | | |   |-Bordetella parapertussis 12822
  | | | |   |-Bordetella pertussis Tohama I
  | | | |-----Burkholderia fungorum LB400
  | | | | | |-Burkholderia mallei ATCC 23344
  | | | | | |---Burkholderia pseudomallei K96243
  | | | | |-----Ralstonia metallidurans CH34
  | | | | | |---Ralstonia eutropha JMP134
  | | | | |---Ralstonia solanacearum GMI1000
  | | | |-----Polaromonas sp. JS666
  | | | |-------Rubrivivax gelatinosus PM1
  | | |-------Thiobacillus denitrificans ATCC 25259
  | | |-------Methylobacillus flagellatus KT
  | | |---------Chromobacterium violaceum ATCC 12472
  | | |   |---Neisseria gonorrhoeae FA 1090
  | | |     |-----Neisseria meningitidis (A) Z2491 (M2677, M4775, M1124, F8229)
  | | |       |---Neisseria meningitidis (B) MC58
  | | |       |---Neisseria meningitidis (B) (unknown strain)
  | | |       |---Neisseria meningitidis (L) (unknown strain)
  | | |       |---Neisseria meningitidis (X) M7575
  | | |-------Nitrosomonas europaea ATCC 19718
  | | |-------Azoarcus sp. EbN1
  | |     |---Dechloromonas aromatica RCB
  | |-----------Bdellovibrio bacteriovorus HD100
  | | | |-------Desulfotalea psychrophila LSv54
  | | | |-------Desulfovibrio desulfuricans G20
  | | | |     |-----Desulfovibrio vulgaris subsp. vulgaris str. Hildenborough
  | | | |-------Geobacter metallireducens
  | | |       |-Geobacter sulfurreducens PCA
  | | |---------Campylobacter coli RM2228
  | |     |   |-Campylobacter jejuni RM1221
  | |     |   |-----Campylobacter jejuni subsp. jejuni NCTC 11168
  | |     |   |-Campylobacter lari RM2100
  | |     |   |-Campylobacter upsaliensis RM3195
  | |     |-----Helicobacter hepaticus ATCC 51449
  | |       | |-Helicobacter pylori 26695
  | |       | |-Helicobacter pylori J99
  | |       |---Wolinella succinogenes DSM 1740
  | |---------Aeromonas hydrophila PPD 11/90, JCM3983
  | | |-------Saccharophagus degradans 14642
  | | |-------Saccharophagus degradans 2-40
  | | | |-----Colwellia psychrerythraea 34H
  | | | |-----Idiomarina loihiensis L2TR
  | | | |-----Shewanella oneidensis MR-1
  | | |-----------Buchnera aphidicola str. APS (Acyrthosiphon pisum)
  | | |   |   |---Buchnera aphidicola str. Bp (Baizongia pistaciae)
  | | |   |   |---Buchnera aphidicola str. Sg (Schizaphis graminum)
  | | |   |---Escherichia coli CFT073
  | | |   | |-Escherichia coli K12
  | | |   | |-Escherichia coli O157:H7
  | | |   | |-Escherichia coli O157:H7 EDL933
  | | |   |-----Erwinia carotovora subsp. atroseptica SCRI1043
  | | |   |-------Photorhabdus luminescens subsp. laumondii TTO1
  | | |   |-------Salmonella enterica subsp. enterica serovar Choleraesuis
  | | |   | |   |---Salmonella enterica subsp. enterica serovar Typhi str. CT18
  | | |   | |     |-Salmonella enterica subsp. enterica serovar Typhi Ty2
  | | |   | |-Salmonella typhimurium LT2
  | | |   |-------Shigella flexneri 2a str. 2457T
  | | |   |     |-Shigella flexneri 2a str. 301
  | | |   |-------Candidatus Blochmannia floridanus
  | | |   |     |-----Candidatus Blochmannia pennsylvanicus str. BPEN
  | | |   |---Wigglesworthia glossinidia endosymbiont of Glossina brevipalpis
  | | |   |---Yersinia pestis biovar Medievalis str. 91001
  | | |     |-Yersinia pestis CO92
  | | |     |-Yersinia pestis KIM
  | | |     |-Yersinia pseudotuberculosis IP 32953
  | | |-------Coxiella burnetii RSA 493
  | | | |-----Legionella pneumophila str. Lens
  | | |     |-Legionella pneumophila str. Paris
  | | |     |-----Legionella pneumophila subsp. pneumophila str. Philadelphia 1
  | | |-------Methylococcus capsulatus str. Bath
  | | |-------Actinobacillus pleuropneumoniae
  | | |   | | |---Actinobacillus pleuropneumoniae serovar 1 str. 4074
  | | |   | | |---Actinobacillus pleuropneumoniae serovar 12 str. 8329
  | | |   | |-Actinobacillus suis SO4
  | | |   |---Haemophilus ducreyi 35000HP
  | | |   | |-Haemophilus influenzae 86-028NP
  | | |   | |-Haemophilus influenzae R2846
  | | |   | |-Haemophilus influenzae R2866
  | | |   | |-Haemophilus influenzae (d) Rd KW20
  | | |   | |-Haemophilus influenzae (f) 700222
  | | |   |-----Haemophilus somnus 129PT
  | | |   |   |-Haemophilus somnus 2336
  | | |   |---Mannheimia succiniciproducens MBEL55E
  | | |   |-------Pasteurella multocida subsp. multocida str. Pm70
  | | |-------Acinetobacter sp. ADP1
  | | | | |---Psychrobacter arcticum 273-4
  | | | |-------Azotobacter vinelandii
  | | |   |-----Pseudomonas aeruginosa PAO1
  | | |     | |-Pseudomonas aeruginosa UCBPP-PA14
  | | |     |---Pseudomonas fluorescens Pf-5
  | | |     | |-Pseudomonas fluorescens PfO-1
  | | |     |---Pseudomonas putida KT2440
  | | |     |---------Pseudomonas syringae pv. syringae B728a
  | | |       |-------Pseudomonas syringae pv. phaseolicola 1448A
  | | |       |-----Pseudomonas syringae pv. tomato str. DC3000
  | | |-----------Francisella tularensis subsp. tularensis SCHU S4
  | | |-------Photobacterium profundum SS9
  | | |   |---------Vibrio cholerae O1 biovar eltor str. N16961
  | | |     |-Vibrio fischeri ES114
  | | |     |-Vibrio parahaemolyticus RIMD 2210633
  | | |     |-Vibrio vulnificus CMCP6
  | | |     |-Vibrio vulnificus YJ016
  | | |-----------Xanthomonas axonopodis pv. citri str. 306
  | |     | |-----Xanthomonas campestris pv. campestris str. 8004
  | |     | |   |-Xanthomonas campestris pv. campestris str. ATCC 33913
  | |     | |-----Xanthomonas oryzae pv. oryzae KACC10331
  | |     |---Xylella fastidiosa 9a5c
  | |       |-Xylella fastidiosa Ann-1
  | |       |-Xylella fastidiosa Dixon
  | |       |-Xylella fastidiosa Temecula1
  | |-----Magnetococcus sp. MC-1
  |---------------Leptospira interrogans serovar Copenhageni str. Fiocruz L1-130
  |     |     |---Leptospira interrogans serovar Lai str. 56601
  |     |-------Borrelia burgdorferi B31
  |       |   |-Borrelia garinii PBi
  |       |---Treponema denticola ATCC 35405
  |         |-----Treponema pallidum subsp. pallidum str. Nichols
  |-----------Thermotoga maritima MSB8
```
